# Supplementary material for: Splatter: simulation of single-cell RNA sequencing data
Source: Genome Biol. 2017 Sep 12;18:174. doi: 10.1186/s13059-017-1305-0 (PMC5596896; doi:10.1186/s13059-017-1305-0)
Supplement: Supplementary file 1 — Diagrams of other simulation models, Splatter comparison output for all datasets, example non-linear gene, dispersion estimate correction, mean-zeros fit, benchmarking, and processing times (PDF 17991 kb) [file 13059_2017_1305_MOESM1_ESM.pdf]

Additional figures

List of additional figures

Additional Figure 1 - Simple simulation model

Additional Figure 2 - Lun simulation model

Additional Figure 3 - Lun 2 simulation model

Additional Figure 4 - Benchmarking (genes)

Additional Figure 5 - Benchmarking (cells)

Additional Figure 6 - Camp dataset comparison

Additional Figure 7 - Klein dataset comparison

Additional Figure 8 - Tung dataset comparison

Additional Figure 9 - Zeisel dataset comparison

Additional Figure 10 - Engel dataset comparison

Additional Figure 11 - Dataset processing times

Additional Figure 12 - MADs heatmap

Additional Figure 13 - Filtered Tung dataset comparison

Additional Figure 14 - Gene goodness-of-fit

Additional Figure 15 - Example non-linear gene

Additional Figure 16 - Dispersion estimation

Additional Figure 17 - Mean-zeros relationship

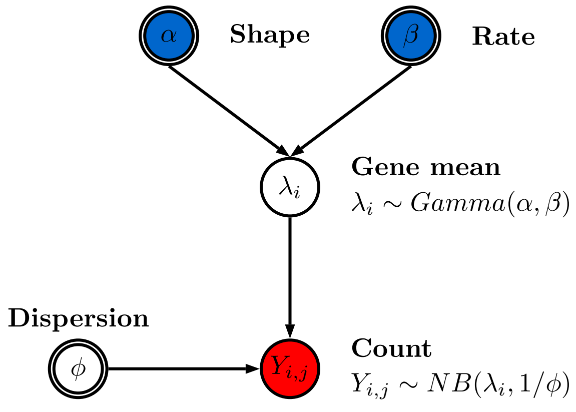

Figure 1: Diagram of the Simple simulation model. Input parameters are indicated with double borders, blue shading shows those that can be estimated from real data. Red shading indicates the final output.

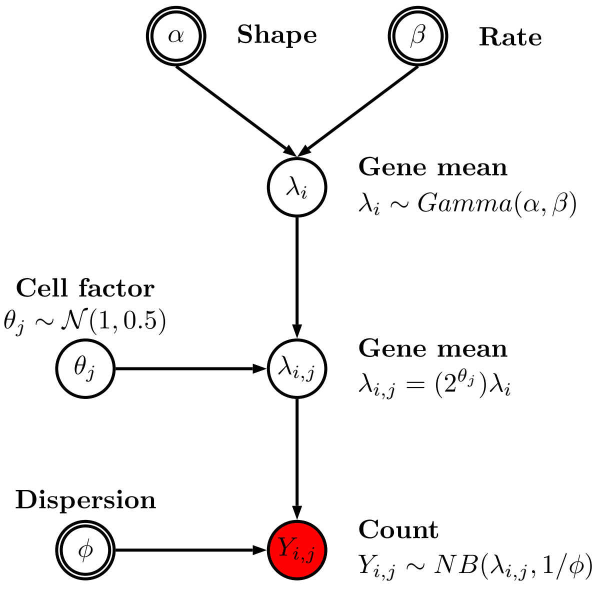

Figure 2: Diagram of the core Lun simulation model. Input parameters are indicated with double borders, blue shading shows those that can be estimated from real data. Red shading indicates the final output.

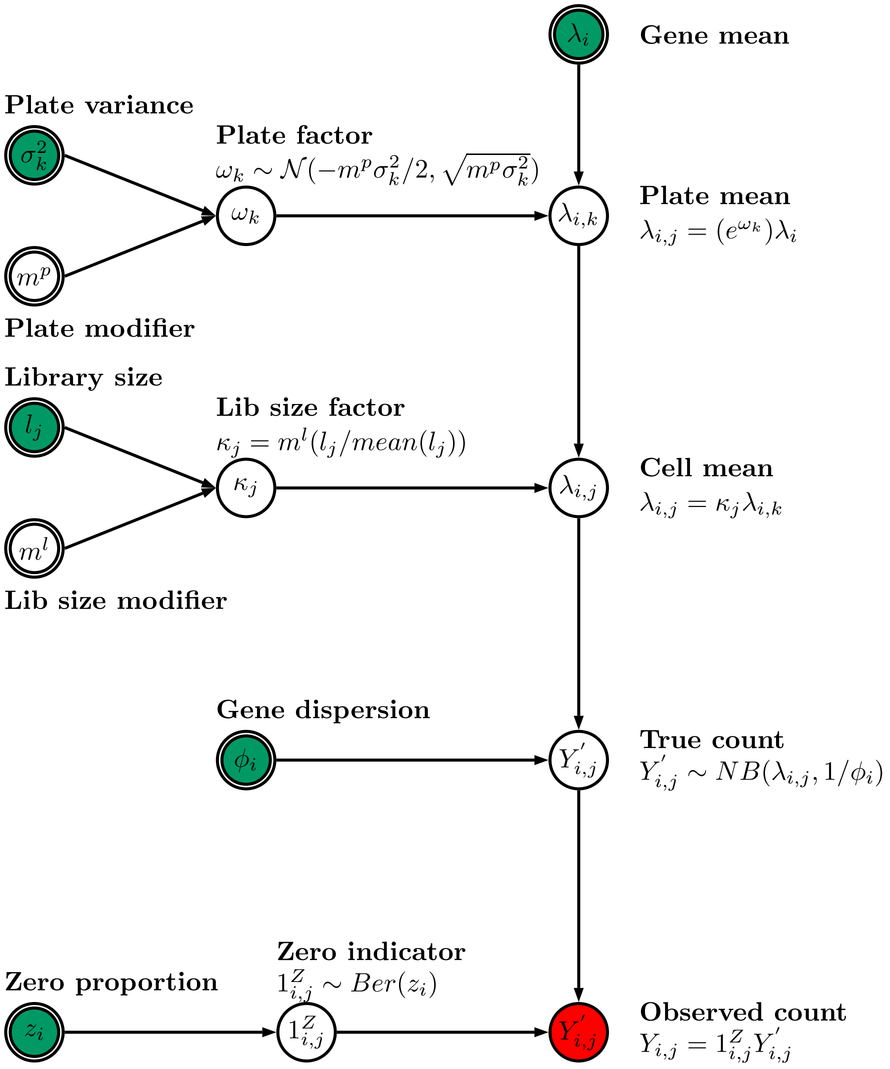

Figure 3: Diagram of the Lun 2 simulation model. Input parameters are indicated with double borders, green shading shows those that can be estimated and sampled from real data. Red shading indicates the final output.

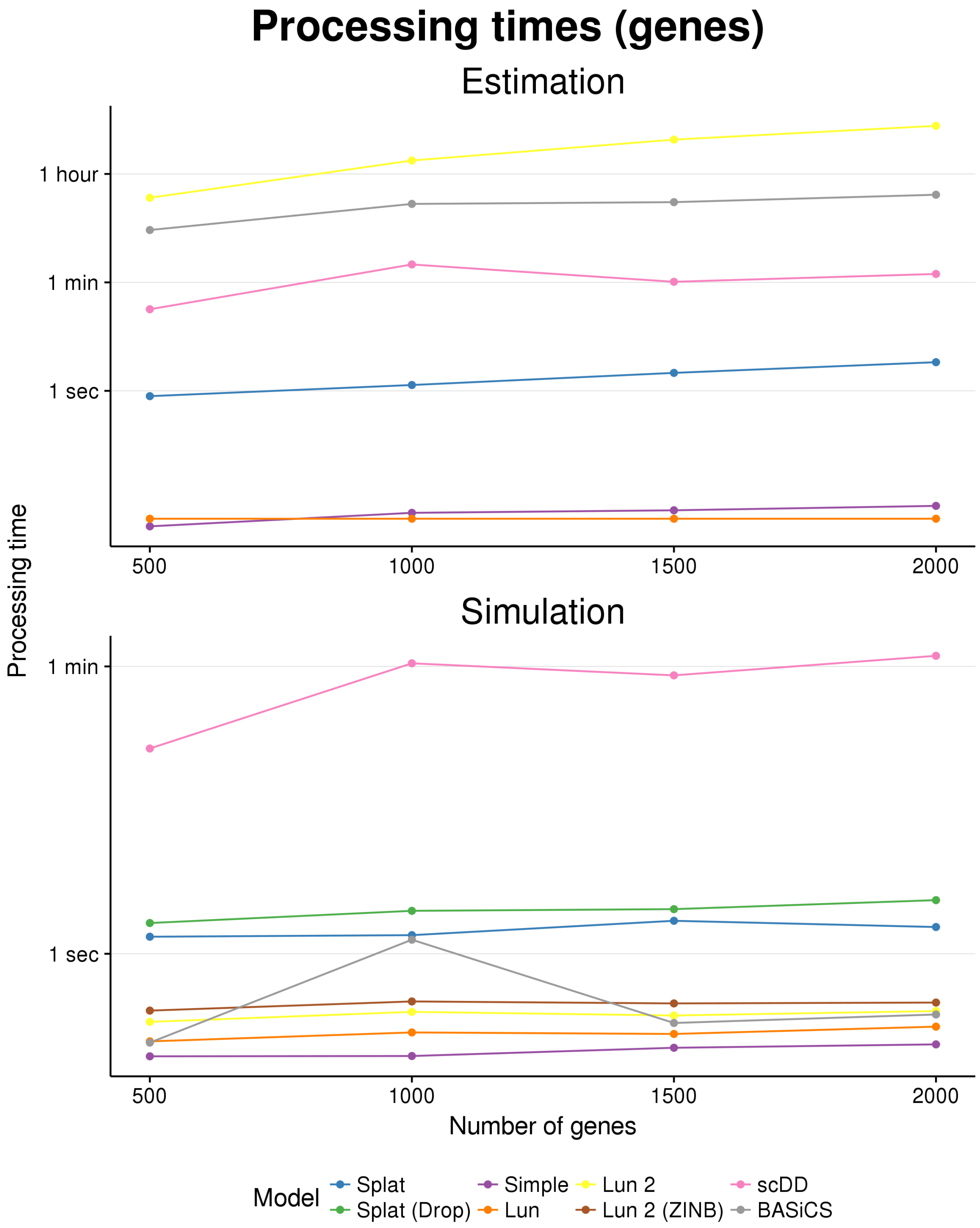

Figure 4: Benchmarking of processing times with incresing numbers of genes. The number of genes in the input dataset is given on the x-axis and the processing time on the y-axis on a log scale. Each simulation is shown in a different colour with the top panel showing the estimation time and the bottom the simulation time. The Splat (Drop) and Lun 2 (ZINB) simulations are not shown in the estimation panel as the parameters for these are estimated at the same time as their regular variants.

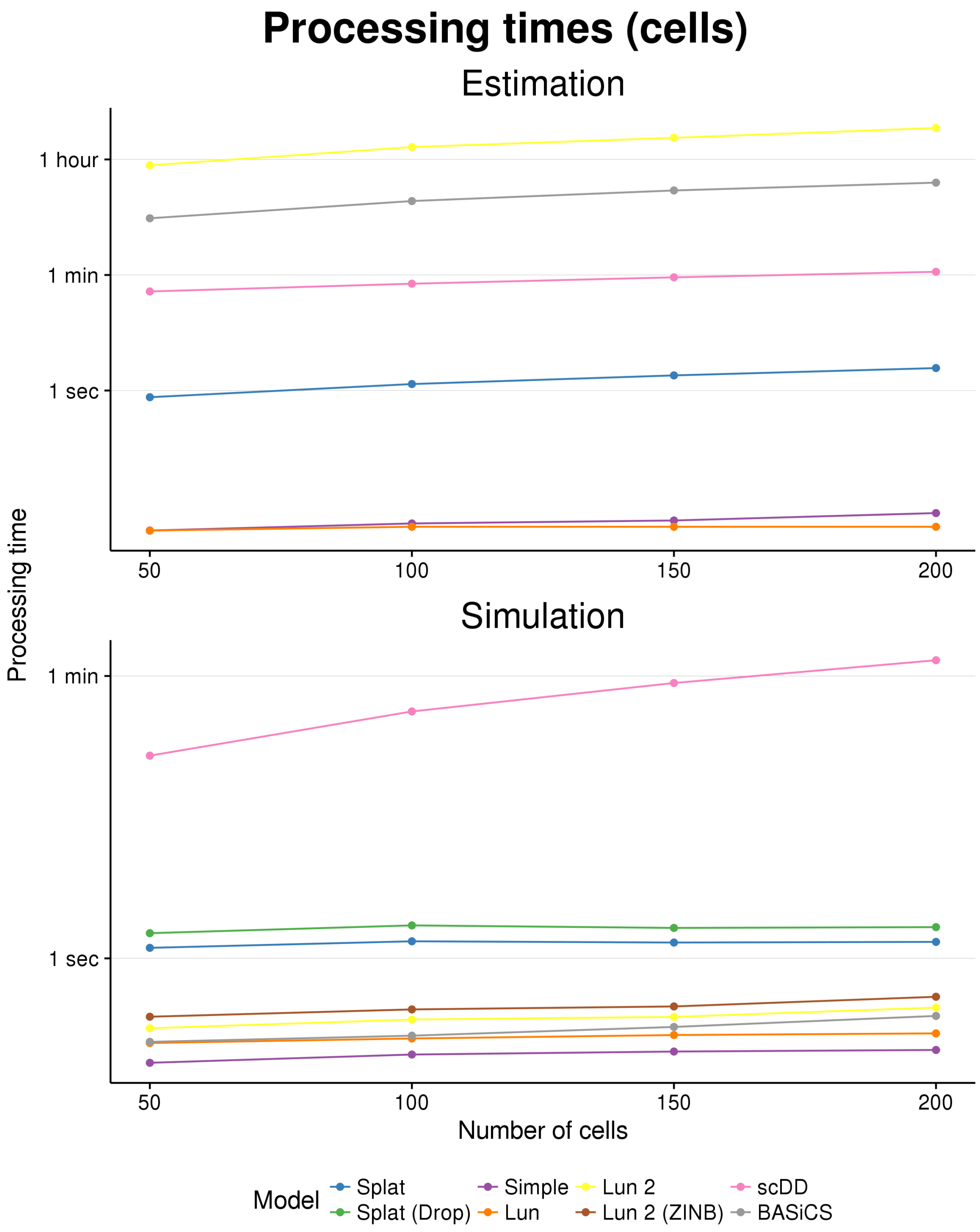

Figure 5: Benchmarking of processing times with incresing numbers of cells. This figure is the same as Additional figure 13 except that the x-axis shows an incresing number of cells rather than genes.

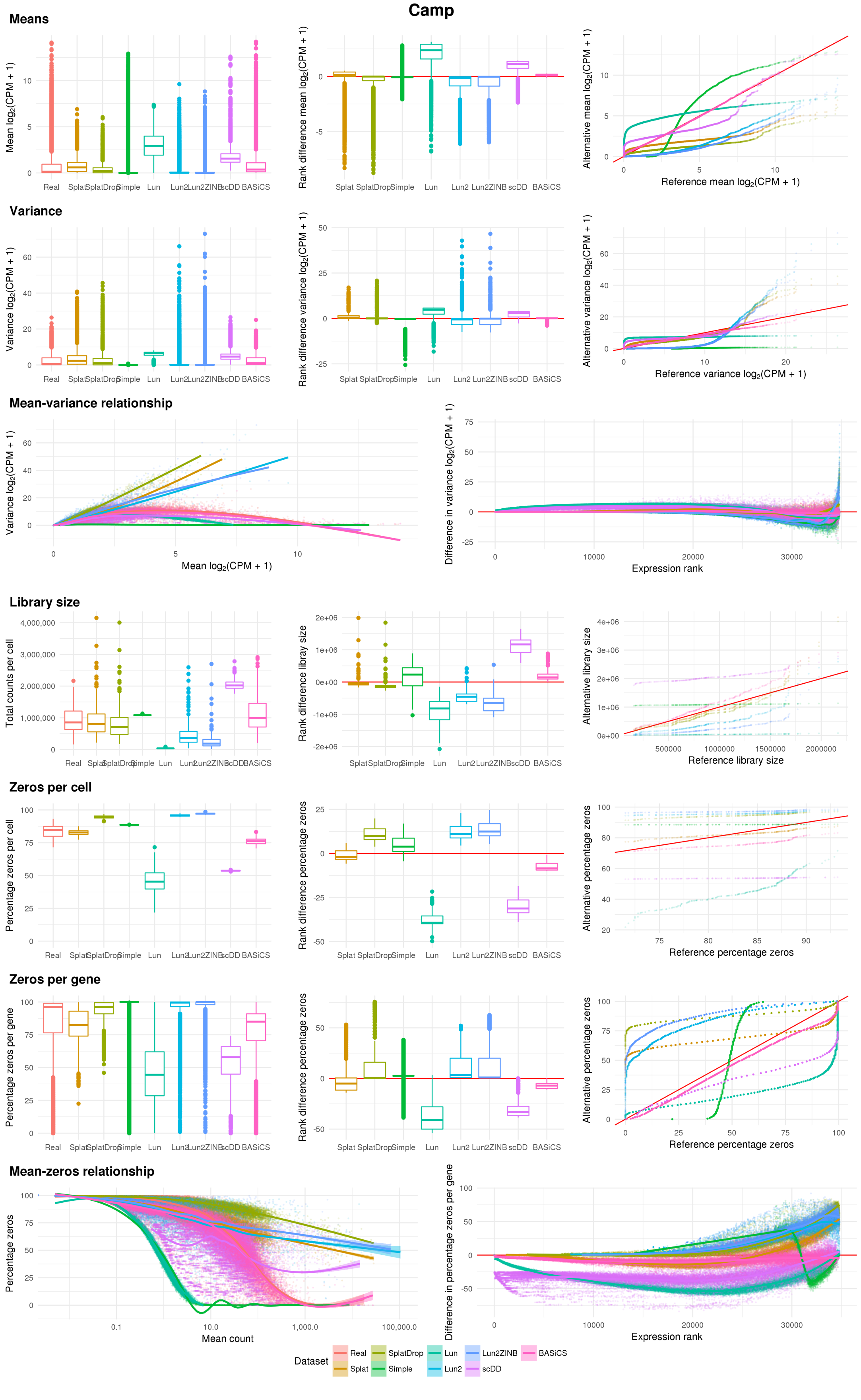

Figure 6: Output of Splatter's comparison functions for simulations based on the Camp dataset.

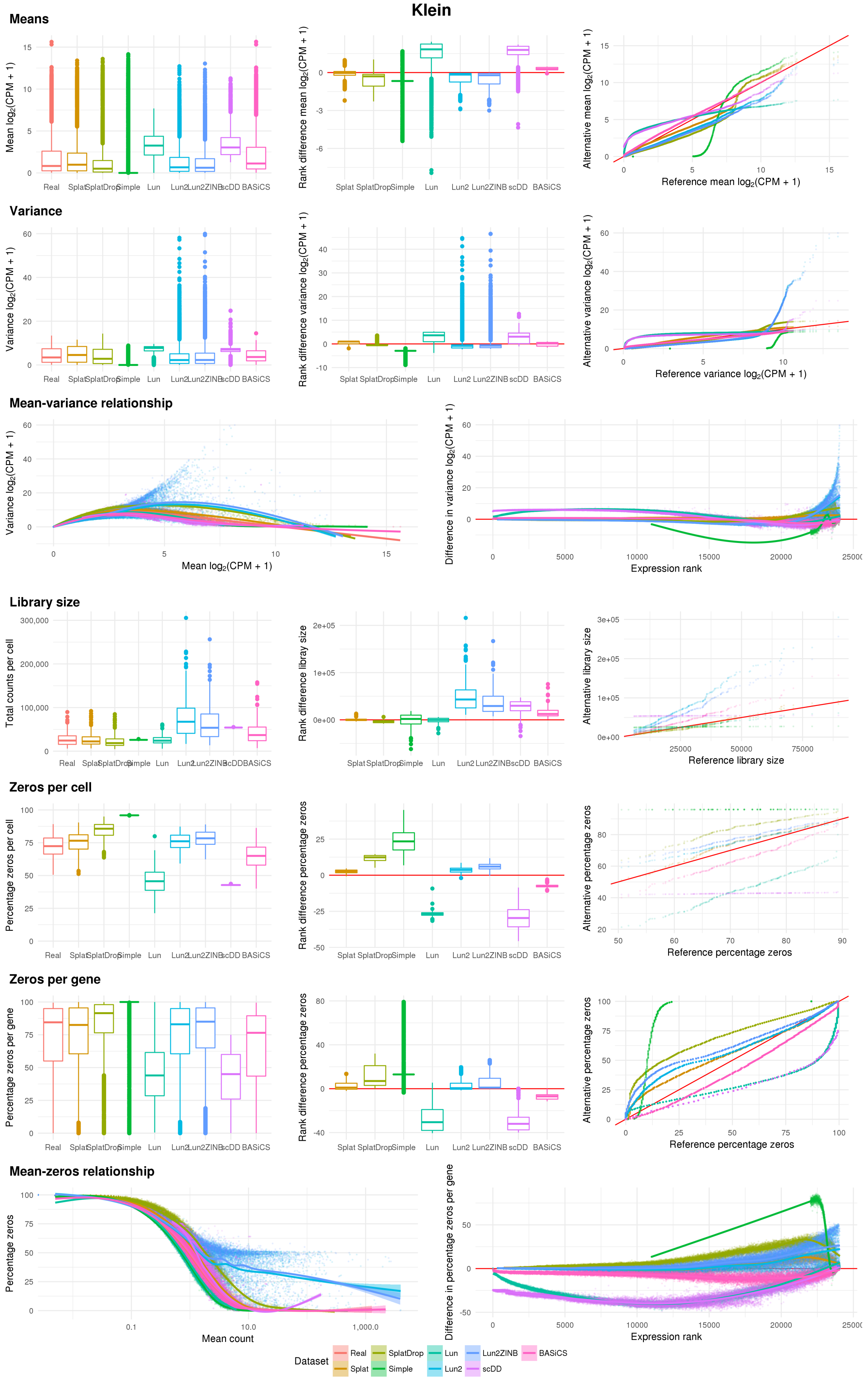

Figure 7: Output of Splatter's comparison functions for simulations based on the Klein dataset.

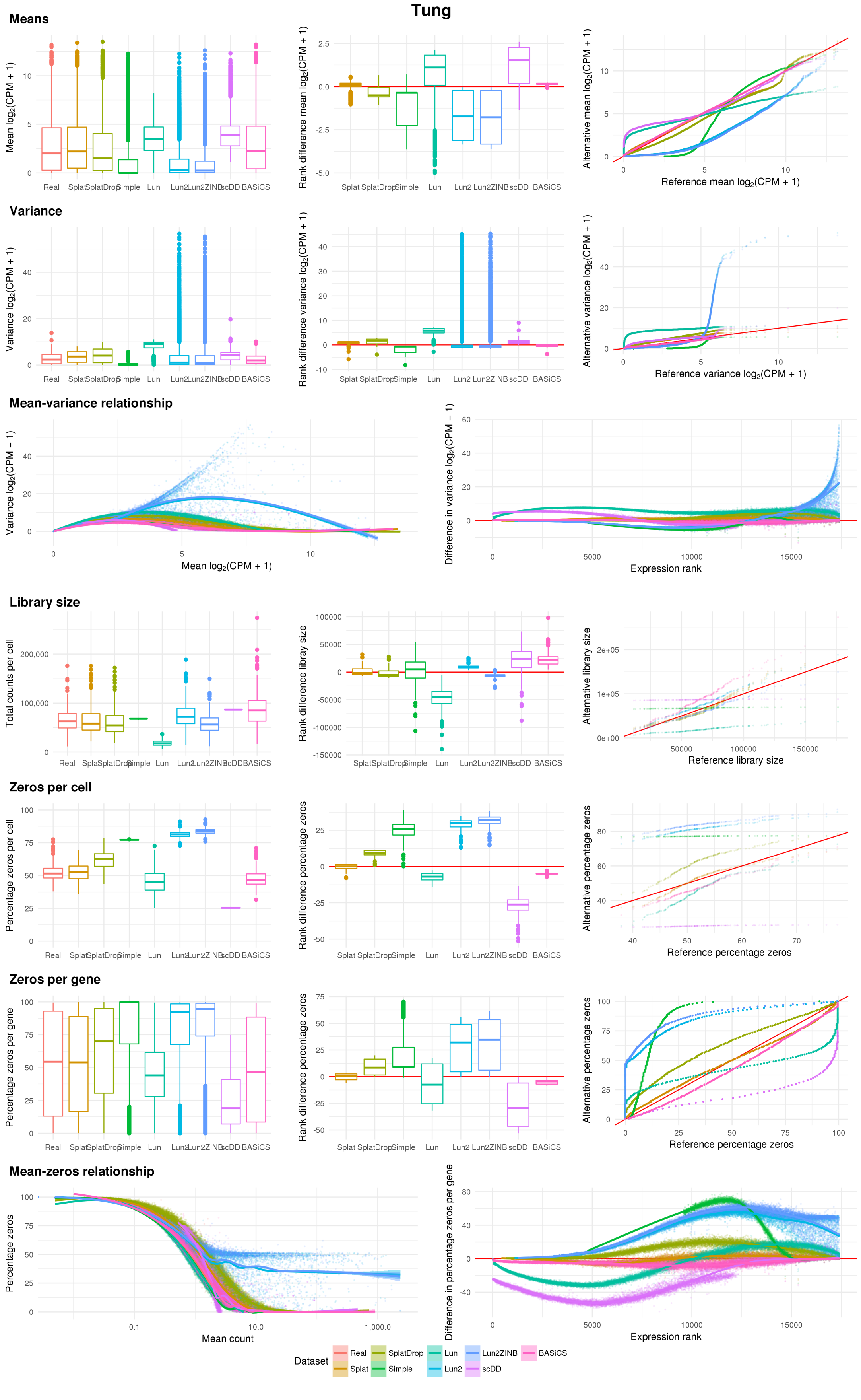

Figure 8: Output of Splatter's comparison functions for simulations based on the Tung dataset.

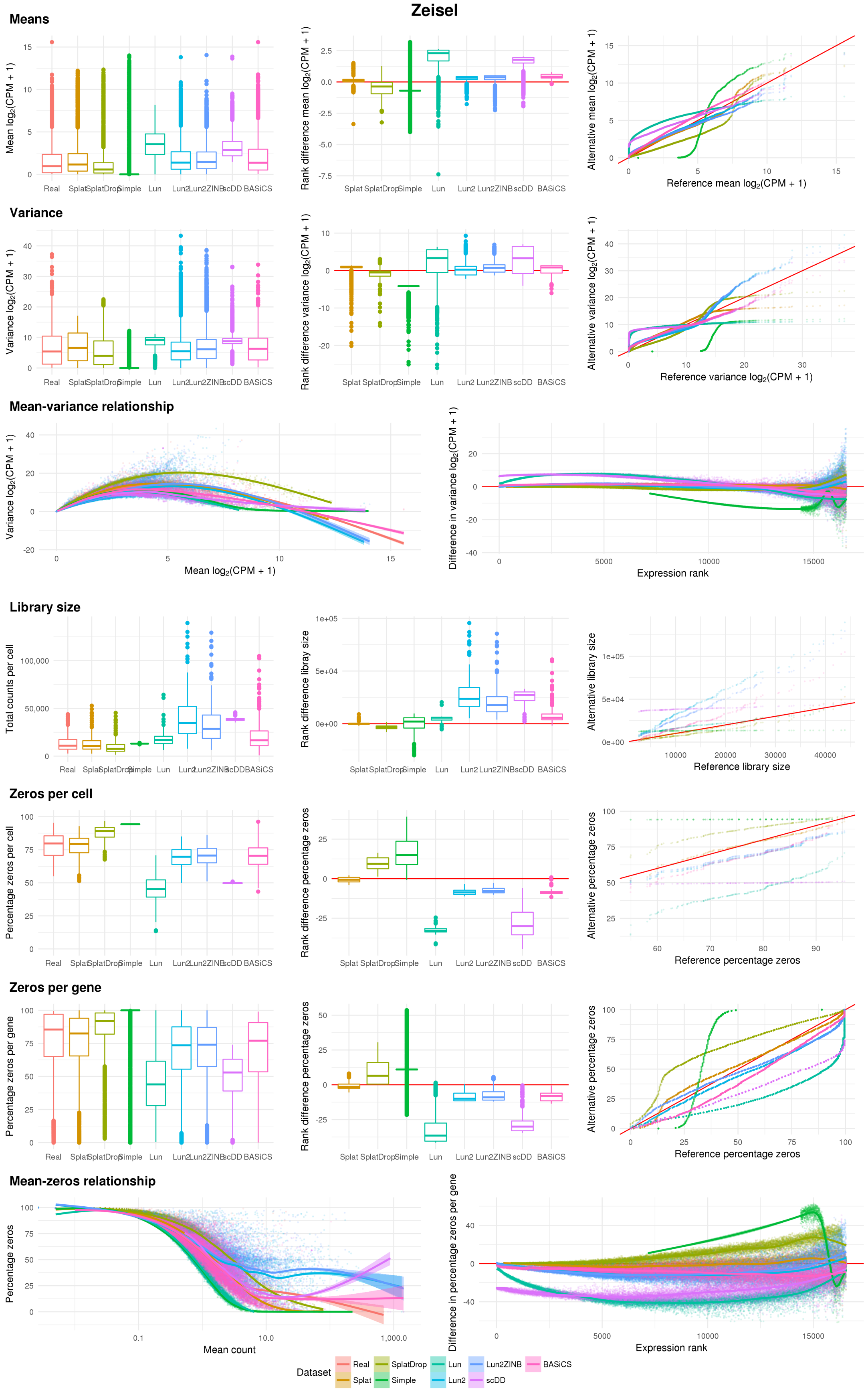

Figure 9: Output of Splatter's comparison functions for simulations based on the Zeisel dataset.

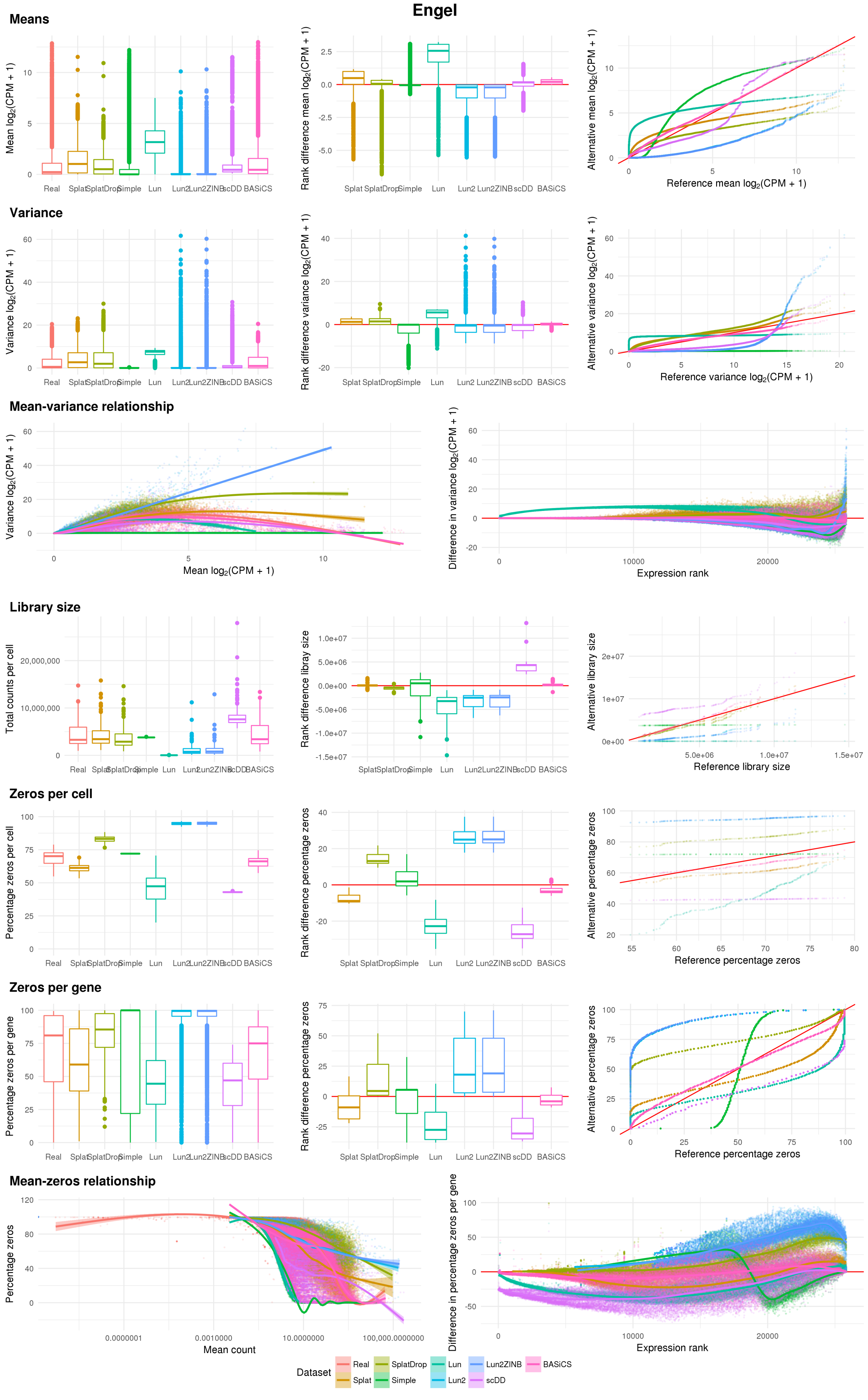

Figure 10: Output of Splatter's comparison functions for simulations based on the Engel dataset.

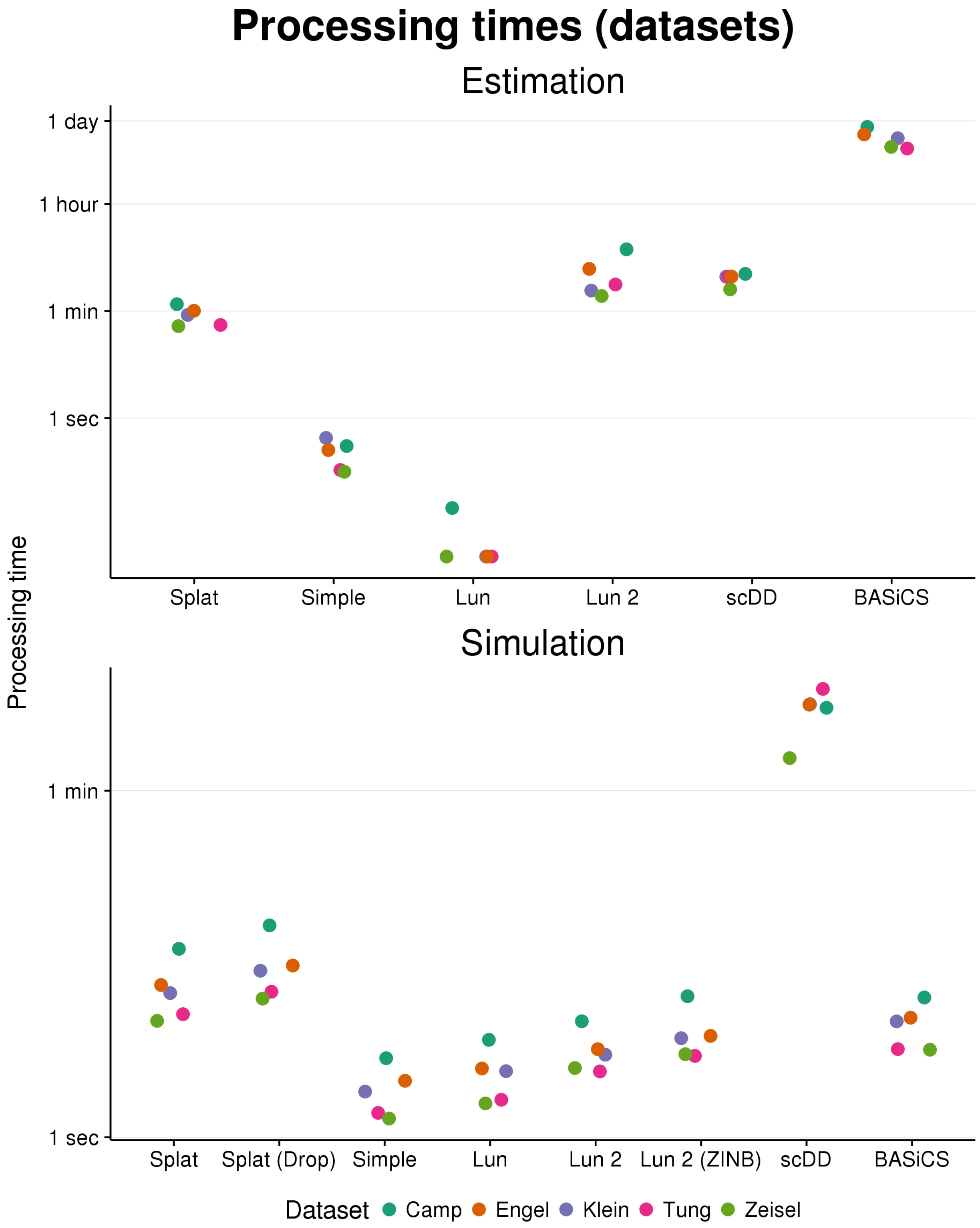

Figure 11: Processing times for the different datasets. The various simulation models are shown on the x-axis and the times they took for estimation (top) and simulation (bottom) are given on the y-axis on a log scale. The scDD estimation, Lun 2 estimation and scDD simulation processes were run on 10 cores, all other processes were run on a single core.

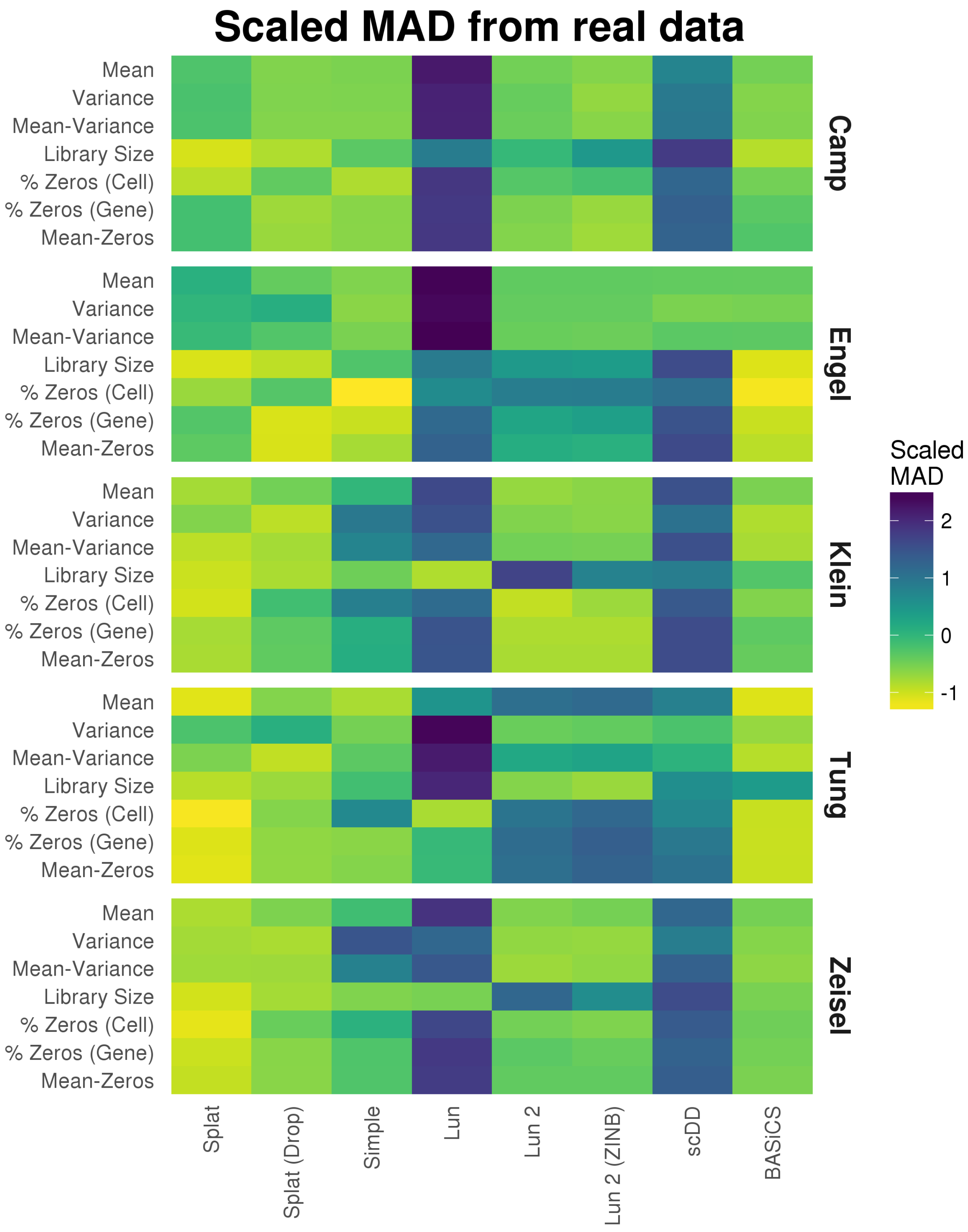

Figure 12: Heatmap of MADs for each simulation and dataset. As the different properties have different scales each row has been scaled by subtracting by the mean and dividing by the standard deviation.

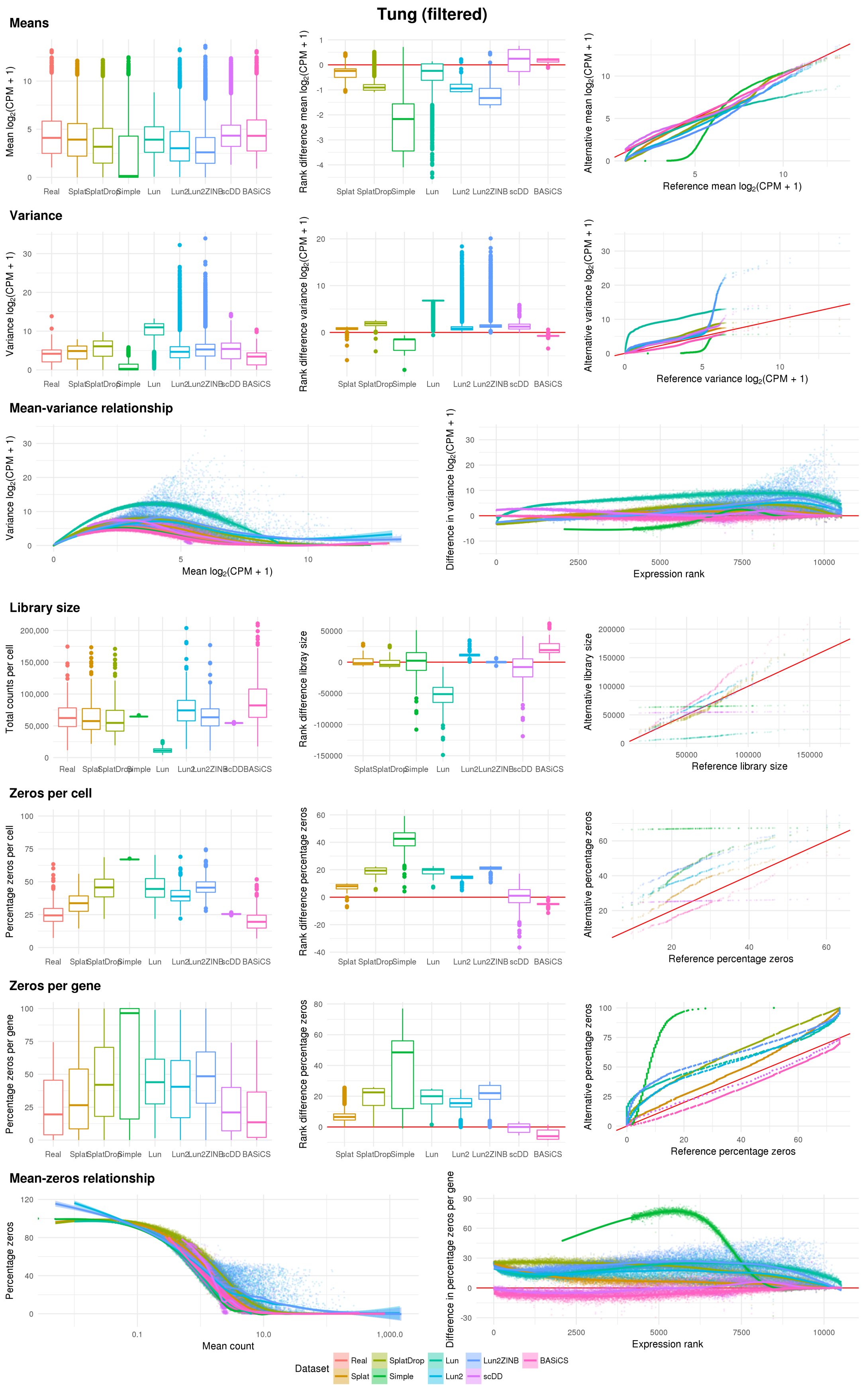

Figure 13: Output of Splatter's comparison functions for simulations based on the Tung dataset after removing all genes with greater than 75 percent zeros.

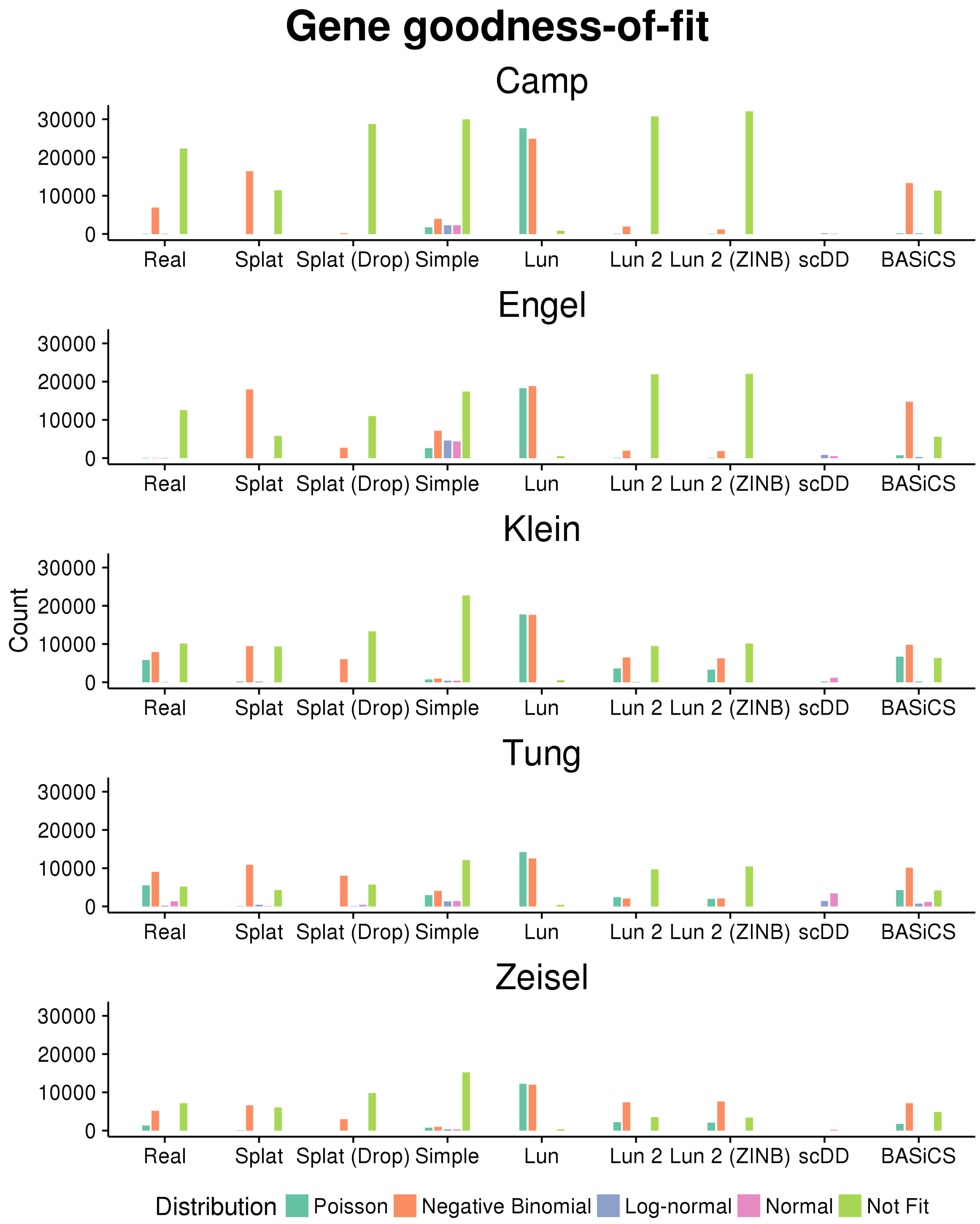

Figure 14: Barplots of gene goodness-of-fit for each dataset. Parameters for the Poisson, negative binomial, log-normal and normal distributions were fit to each gene. A chi-squared goodness-of-fit test was then used to see if the counts for that gene could be expected to come from the fitted distributions. The height of the bars show the number of genes for which a given distribution was not rejected ($p > 0.01$), as well as the number of genes for which fitting failed for all distributions.

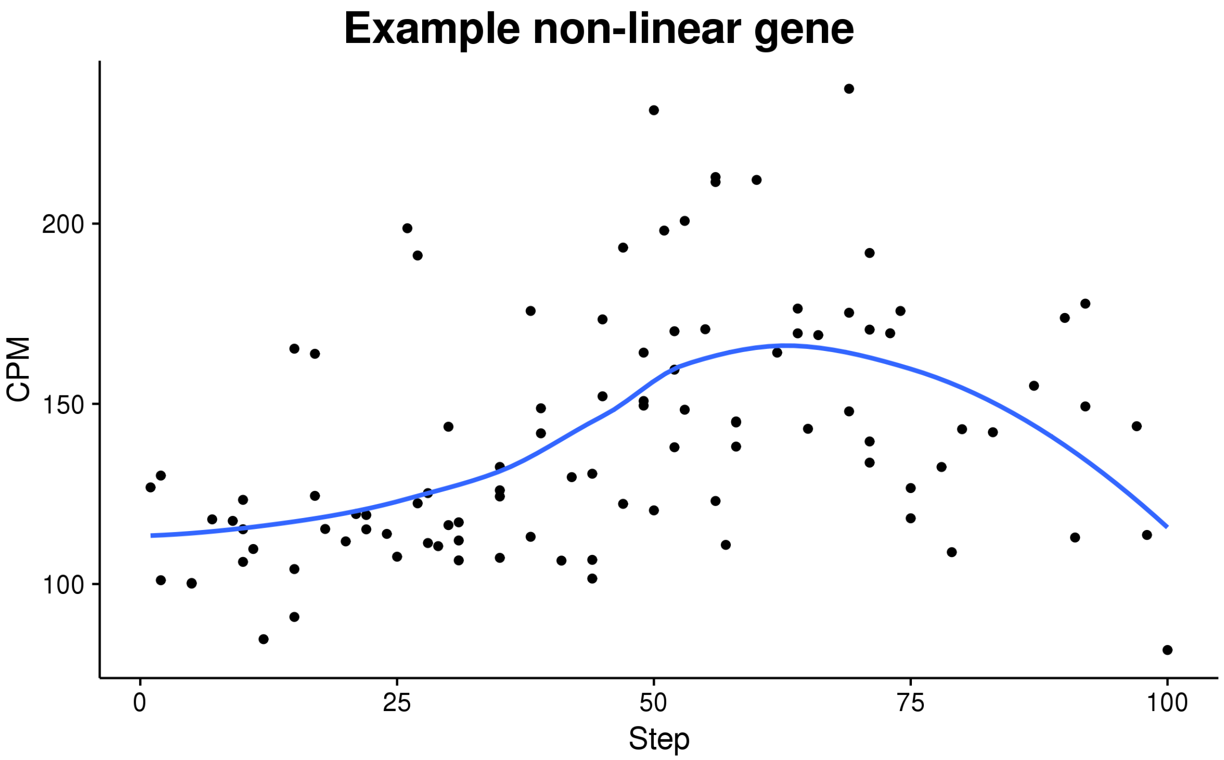

Figure 15: Example of a simulated gene that changes expression in a non-linear way over the course of a path. Each point is a cell with the x-axis showing progression along the path and the y-axis the simulated expression level. The blue line shows a LOESS fit.

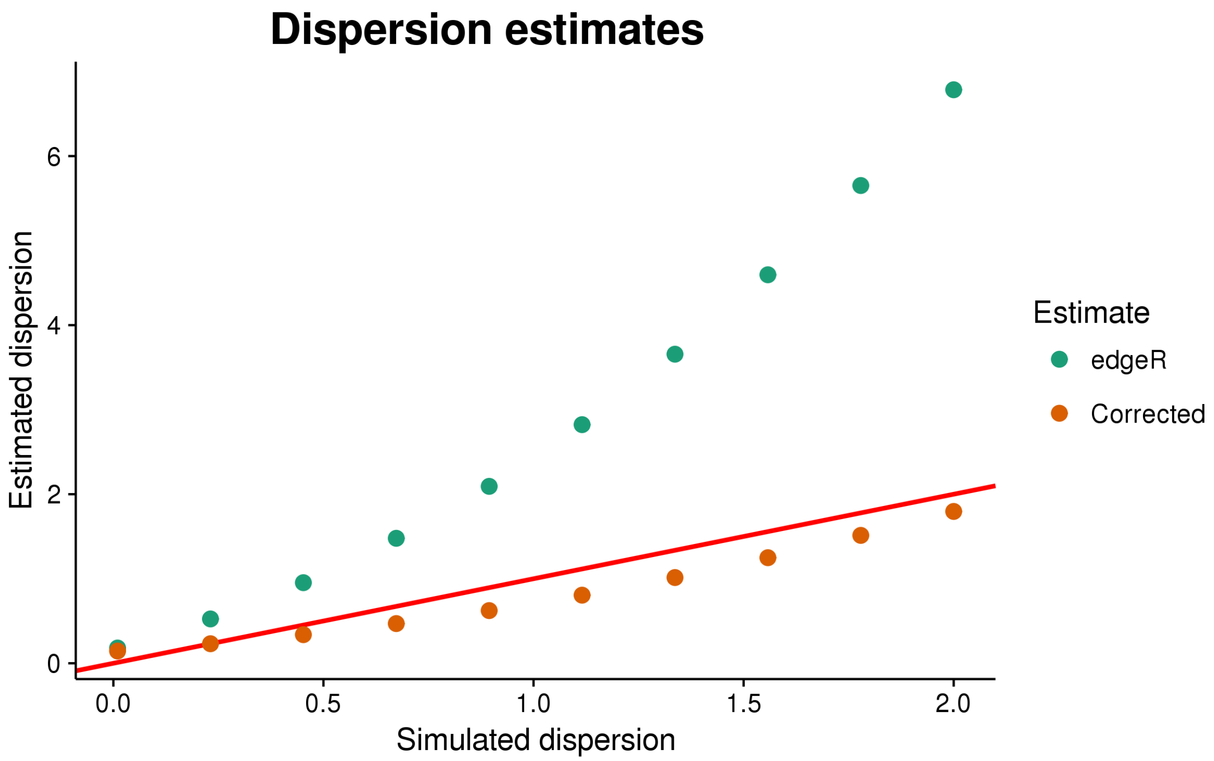

Figure 16: Correction of edgeR dispersion estimates. Scatter plot of estimated dispersions against the true simulated values. Estimates of common dispersion obtained from edgeR (green) can be inflated for single-cell data. The Splat simulation uses a linearly corrected value (orange) in its estimation procedure. The red line shows the true values.

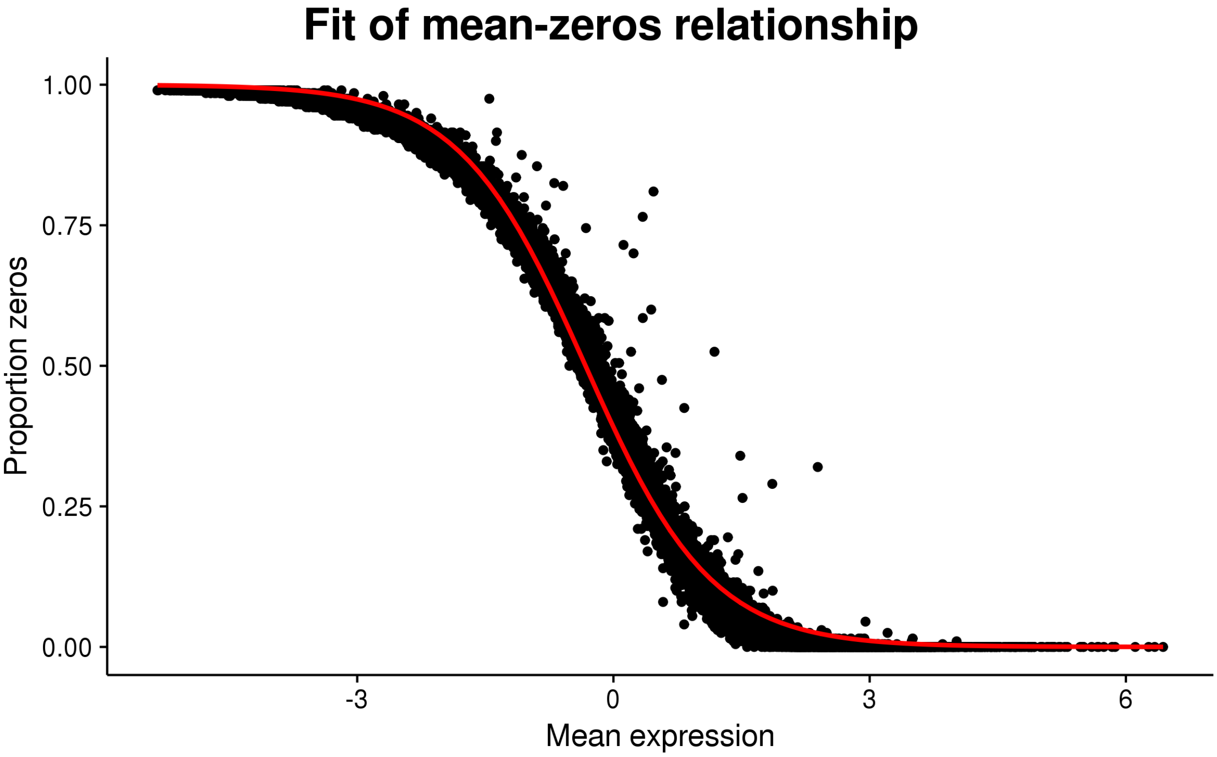

Figure 17: Fitting the mean-zeros relationship. Points show genes in the Tung dataset and the red line is a logistic function fitted to the data using Splat's estimation procedure.
