# Supplementary material for: Splatter: simulation of single-cell RNA sequencing data
Source: Genome Biol. 2017 Sep 12;18:174. doi: 10.1186/s13059-017-1305-0 (PMC5596896; doi:10.1186/s13059-017-1305-0)
Supplement: Supplementary file 3 — Session information. Details of the R environment and packages used for analysis. (PDF 118 kb) [file 13059_2017_1305_MOESM3_ESM.pdf]

R Session Information

2017-07-20

Code version: 905b291f81259313ee06d326524c31541d622055

Session information

Details of the R environment and packages used for analysis.

```
##  setting  value
##  version  R version 3.4.0 (2017-04-21)
##  system   x86_64, linux-gnu
##  ui       X11
##  language (EN)
##  collate  en_US.UTF-8
##  tz       <NA>
##  date     2017-07-20
##
##  package             * version  date
##  AnnotationDbi         1.38.1   2017-06-05
##  assertthat            0.2.0    2017-04-11
##  backports             1.1.0    2017-05-22
##  base                * 3.4.0    2017-05-30
##  beeswarm              0.2.3    2016-04-25
##  bindr                 0.1      2016-11-13
##  bindrcpp            * 0.2      2017-06-17
##  Biobase             * 2.36.2   2017-05-31
##  BiocGenerics        * 0.22.0   2017-05-31
##  BiocParallel        * 1.10.1   2017-05-31
##  biomaRt               2.32.1   2017-06-13
##  bit                   1.1-12   2014-04-09
##  bit64                 0.9-7    2017-05-08
##  bitops                1.0-6    2013-08-17
##  blob                  1.1.0    2017-06-17
##  broom                 0.4.2    2017-02-13
##  caTools               1.17.1   2014-09-10
##  cellranger            1.1.0    2016-07-27
##  checkmate             1.8.3    2017-07-03
##  class                 7.3-14   2015-08-30
##  clues               * 0.5.9    2016-10-14
##  cluster               2.0.6    2017-03-10
##  codetools             0.2-15   2016-10-05
##  colorspace            1.3-2    2016-12-14
##  compiler              3.4.0    2017-05-30
##  cowplot             * 0.7.0    2016-10-28
##  data.table            1.10.4   2017-02-01
##  datasets            * 3.4.0    2017-05-30
##  DBI                   0.7      2017-06-18
##  DelayedArray          0.2.7    2017-06-05
##  DEoptimR              1.0-8    2016-11-19
##  devtools            * 1.13.2   2017-06-02
```

```
##  digest                0.6.12   2017-01-27
##  doParallel            1.0.10   2015-10-14
##  doRNG                 1.6.6    2017-04-10
##  dplyr               * 0.7.1    2017-06-22
##  e1071                 1.6-8    2017-02-02
##  edgeR                 3.18.1   2017-06-14
##  evaluate              0.10     2016-10-11
##  forcats               0.2.0    2017-01-23
##  foreach               1.4.3    2015-10-13
##  foreign               0.8-68   2017-04-24
##  gdata                 2.18.0   2017-06-06
##  GenomeInfoDb          1.12.2   2017-06-13
##  GenomeInfoDbData      0.99.0   2017-05-31
##  GenomicRanges         1.28.3   2017-05-31
##  ggbeeswarm            0.5.3    2016-12-01
##  ggplot2             * 2.2.1    2016-12-30
##  glue                  1.1.1    2017-06-21
##  gplots                3.0.1    2016-03-30
##  graphics            * 3.4.0    2017-05-30
##  grDevices           * 3.4.0    2017-05-30
##  grid                  3.4.0    2017-05-30
##  gridExtra             2.2.1    2016-02-29
##  gtable                0.2.0    2016-02-26
##  gtools                3.5.0    2015-05-29
##  haven                 1.0.0    2016-09-23
##  hms                   0.3      2016-11-22
##  htmltools             0.3.6    2017-04-28
##  httpuv                1.3.3    2015-08-04
##  httr                  1.2.1    2016-07-03
##  IRanges               2.10.2   2017-05-31
##  iterators             1.0.8    2015-10-13
##  jsonlite              1.5      2017-06-01
##  KernSmooth            2.23-15  2015-06-29
##  knitr               * 1.16     2017-05-18
##  lattice               0.20-35  2017-03-25
##  lazyeval              0.2.0    2016-06-12
##  limma                 3.32.2   2017-06-14
##  locfit                1.5-9.1  2013-04-20
##  lubridate             1.6.0    2016-09-13
##  magrittr            * 1.5      2014-11-22
##  Matrix                1.2-10   2017-04-28
##  matrixStats           0.52.2   2017-04-14
##  memoise               1.1.0    2017-04-21
##  methods             * 3.4.0    2017-05-30
##  mime                  0.5      2016-07-07
##  mnormt                1.5-5    2016-10-15
##  modelr                0.1.0    2016-08-31
##  munsell               0.4.3    2016-02-13
##  mvtnorm               1.0-6    2017-03-02
##  nlme                  3.1-131  2017-02-06
##  parallel            * 3.4.0    2017-05-30
##  pcaPP                 1.9-72   2017-06-27
##  pheatmap              1.0.8    2015-12-11
##  pkgconfig             2.0.1    2017-03-21
```

```
##  pkgmaker               0.22     2014-05-14
##  plyr                   1.8.4    2016-06-08
##  psych                  1.7.5    2017-05-03
##  purrr                * 0.2.2.2  2017-05-11
##  R6                     2.2.2    2017-06-17
##  RColorBrewer           1.1-2    2014-12-07
##  Rcpp                   0.12.12  2017-07-15
##  RCurl                  1.95-4.8 2016-03-01
##  readr                * 1.1.1    2017-05-16
##  readxl                 1.0.0    2017-04-18
##  registry               0.3      2015-07-08
##  reshape2               1.4.2    2016-10-22
##  rhdf5                  2.20.0   2017-05-31
##  rjson                  0.2.15   2014-11-03
##  rlang                  0.1.1    2017-05-18
##  rmarkdown            * 1.6      2017-06-15
##  rngtools               1.2.4    2014-03-06
##  robustbase             0.92-7   2016-12-09
##  ROCR                   1.0-7    2015-03-26
##  rprojroot              1.2      2017-01-16
##  rrcov                  1.4-3    2016-09-06
##  RSQLite                2.0      2017-06-19
##  rstudioapi             0.6      2016-06-27
##  rvest                  0.3.2    2016-06-17
##  S4Vectors              0.14.3   2017-06-05
##  SC3                  * 1.4.2    2017-07-13
##  scales                 0.4.1    2016-11-09
##  scater               * 1.4.0    2017-06-14
##  shiny                  1.0.3    2017-04-26
##  shinydashboard         0.6.1    2017-06-14
##  splatter             * 1.1.2    2017-07-16
##  stats                * 3.4.0    2017-05-30
##  stats4                 3.4.0    2017-05-30
##  stringi                1.1.5    2017-04-07
##  stringr                1.2.0    2017-02-18
##  SummarizedExperiment   1.6.3    2017-05-31
##  tibble               * 1.3.3    2017-05-28
##  tidyr                * 0.6.3    2017-05-15
##  tidyverse            * 1.1.1    2017-01-27
##  tools                  3.4.0    2017-05-30
##  tximport               1.4.0    2017-05-31
##  utils                * 3.4.0    2017-05-30
##  vipor                  0.4.5    2017-03-22
##  viridis                0.4.0    2017-03-27
##  viridisLite            0.2.0    2017-03-24
##  withr                  1.0.2    2016-06-20
##  WriteXLS               4.0.0    2015-12-07
##  XML                    3.98-1.9 2017-06-19
##  xml2                   1.1.1    2017-01-24
##  xtable                 1.8-2    2016-02-05
##  XVector                0.16.0   2017-05-31
##  yaml                   2.1.14   2016-11-12
##  zlibbioc               1.22.0   2017-05-31
##  source
```

```
##  Bioconductor
##  CRAN (R 3.4.0)
##  CRAN (R 3.4.0)
##  local
##  CRAN (R 3.4.0)
##  CRAN (R 3.4.0)
##  CRAN (R 3.4.0)
##  Bioconductor
##  Bioconductor
##  Bioconductor
##  Bioconductor
##  CRAN (R 3.4.0)
##  CRAN (R 3.4.0)
##  CRAN (R 3.4.0)
##  CRAN (R 3.4.0)
##  CRAN (R 3.4.0)
##  CRAN (R 3.4.0)
##  CRAN (R 3.4.0)
##  CRAN (R 3.4.0)
##  CRAN (R 3.4.0)
##  CRAN (R 3.4.0)
##  CRAN (R 3.4.0)
##  CRAN (R 3.4.0)
##  CRAN (R 3.4.0)
##  local
##  CRAN (R 3.4.0)
##  CRAN (R 3.4.0)
##  local
##  CRAN (R 3.4.0)
##  Bioconductor
##  CRAN (R 3.4.0)
##  CRAN (R 3.4.0)
##  CRAN (R 3.4.0)
##  CRAN (R 3.4.0)
##  CRAN (R 3.4.0)
##  CRAN (R 3.4.0)
##  CRAN (R 3.4.0)
##  Bioconductor
##  CRAN (R 3.4.0)
##  CRAN (R 3.4.0)
##  CRAN (R 3.4.0)
##  CRAN (R 3.4.0)
##  CRAN (R 3.4.0)
##  Bioconductor
##  Bioconductor
##  Bioconductor
##  CRAN (R 3.4.0)
##  CRAN (R 3.4.0)
##  CRAN (R 3.4.0)
##  CRAN (R 3.4.0)
##  local
##  local
##  local
##  CRAN (R 3.4.0)
```

```
##  CRAN (R 3.4.0)
##  CRAN (R 3.4.0)
##  CRAN (R 3.4.0)
##  CRAN (R 3.4.0)
##  CRAN (R 3.4.0)
##  CRAN (R 3.4.0)
##  CRAN (R 3.4.0)
##  Bioconductor
##  CRAN (R 3.4.0)
##  CRAN (R 3.4.0)
##  CRAN (R 3.4.0)
##  CRAN (R 3.4.0)
##  CRAN (R 3.4.0)
##  CRAN (R 3.4.0)
##  Bioconductor
##  CRAN (R 3.4.0)
##  CRAN (R 3.4.0)
##  CRAN (R 3.4.0)
##  CRAN (R 3.4.0)
##  CRAN (R 3.4.0)
##  CRAN (R 3.4.0)
##  local
##  CRAN (R 3.4.0)
##  CRAN (R 3.4.0)
##  CRAN (R 3.4.0)
##  CRAN (R 3.4.0)
##  CRAN (R 3.4.0)
##  CRAN (R 3.4.0)
##  local
##  CRAN (R 3.4.0)
##  CRAN (R 3.4.0)
##  CRAN (R 3.4.0)
##  CRAN (R 3.4.0)
##  CRAN (R 3.4.0)
##  CRAN (R 3.4.0)
##  CRAN (R 3.4.0)
##  CRAN (R 3.4.0)
##  CRAN (R 3.4.0)
##  cran (@0.12.12)
##  CRAN (R 3.4.0)
##  CRAN (R 3.4.0)
##  CRAN (R 3.4.0)
##  CRAN (R 3.4.0)
##  CRAN (R 3.4.0)
##  Bioconductor
##  CRAN (R 3.4.0)
##  CRAN (R 3.4.0)
##  CRAN (R 3.4.0)
##  CRAN (R 3.4.0)
##  CRAN (R 3.4.0)
##  CRAN (R 3.4.0)
##  cran (@1.2)
##  CRAN (R 3.4.0)
##  CRAN (R 3.4.0)
```

```
## CRAN (R 3.4.0)
## CRAN (R 3.4.0)
## Bioconductor
## Bioconductor
## CRAN (R 3.4.0)
## Bioconductor
## CRAN (R 3.4.0)
## CRAN (R 3.4.0)
## Github (Oshlack/splatter@5df5a7c)
## local
## local
## CRAN (R 3.4.0)
## CRAN (R 3.4.0)
## Bioconductor
## CRAN (R 3.4.0)
## CRAN (R 3.4.0)
## CRAN (R 3.4.0)
## local
## Bioconductor
## local
## CRAN (R 3.4.0)
## CRAN (R 3.4.0)
## CRAN (R 3.4.0)
## CRAN (R 3.4.0)
## CRAN (R 3.4.0)
## CRAN (R 3.4.0)
## CRAN (R 3.4.0)
## CRAN (R 3.4.0)
## Bioconductor
## CRAN (R 3.4.0)
## Bioconductor
```
